# Nucleoredoxin-Dependent Targets and Processes in Neuronal Cells

**DOI:** 10.1155/2018/4829872

**Published:** 2018-11-21

**Authors:** Claudia Urbainsky, Rolf Nölker, Marcel Imber, Adrian Lübken, Jörg Mostertz, Falko Hochgräfe, José R. Godoy, Eva-Maria Hanschmann, Christopher Horst Lillig

**Affiliations:** ^1^The Institute for Medical Biochemistry and Molecular Biology, University Medicine, University of Greifswald, Germany; ^2^Competence Center Functional Genomics, Junior Research Group Pathoproteomics, University of Greifswald, Germany; ^3^Institute for Biology-Microbiology, Freie Universität Berlin, Germany; ^4^Faculty of Biomedical Sciences, Ross University School of Veterinary Medicine, Basseterre, Saint Kitts and Nevis; ^5^Department of Neurology, Medical Faculty, Heinrich-Heine University Düsseldorf, Germany

## Abstract

Nucleoredoxin (Nrx) is an oxidoreductase of the thioredoxin family of proteins. It was shown to act as a signal transducer in some pathways; however, so far, no comprehensive analysis of its regulated substrates and functions was available. Here, we used a combination of two different strategies to fill this gap. First, we analyzed the thiol-redox state of the proteome of SH-SY5Y neuroblastoma cells depleted of Nrx compared to control cells using a differential thiol-labeling technique and quantitative mass spectrometry. 171 proteins were identified with an altered redox state; 161 of these were more reduced in the absence of Nrx. This suggests functions of Nrx in the oxidation of protein thiols. Second, we utilized the active site mutant Cys208Ser of Nrx, which stabilizes a mixed disulfide intermediate with its substrates and therefore trapped interacting proteins from the mouse brain (identifying 1710 proteins) and neuronal cell culture extracts (identifying 609 proteins). Profiling of the affected biological processes and molecular functions in cells of neuronal origin suggests numerous functions of Nrx in the redox regulation of metabolic pathways, cellular morphology, and signal transduction. These results characterize Nrx as a cellular oxidase that itself may be oxidized by the formation of disulfide relays with peroxiredoxins.

## 1. Introduction

Redox signaling constitutes an essential mechanism for the regulation of protein function within specific, rapid, and highly regulated signaling cascades, comparable to de-/phosphorylation. Specific oxidative modifications of proteins, for instance, the formation of allosteric disulfides, are often attributed to the action of reactive oxygen or nitrogen species, most of all hydrogen peroxide (H_2_O_2_). Due to the low rate constants of H_2_O_2_ with most protein thiols, however, this oxidation as well as the reduction of disulfides requires catalysis by specific enzymes [1, 2].

Nucleoredoxin (Nrx) is a member of the thioredoxin (Trx) family of proteins. This family includes various oxidoreductases, such as Trxs, glutaredoxins (Grxs), peroxiredoxins (Prxs), and protein disulfide isomerases (PDIs) that catalyze cellular redox signaling [3]. All these proteins share a common structural motif, the so-called Trx fold [4]. The Nrx gene (NXN) encodes a protein of 48 kDa and is ubiquitously expressed [5]. Despite its name, the protein is localized in both the cytosol and the nucleus [5, 6]. The Nrx protein contains three Trx domains organized in a structure similar to those of PDIs that contain three to four Trx domains. The N- and C-terminal domains of Nrx share a high similarity to the b′ domains of PDIs and lack a redox active center. The central domain, however, contains the dithiol active site motif Cys-Pro-Pro-Cys and was shown to be active in the insulin reduction assay [6]. The function of the PDI-like domains in Nrx is unclear; they may be important for substrate recognition, i.e., specific protein-protein interactions.

Up to now, no comprehensive identification of potential Nrx substrates has been presented and only scattered information on the function of the protein is available. Two interaction partners that have been identified are phosphofructokinase 1 and protein phosphatase 2A [7, 8]. The activity of both proteins is affected through direct interactions. Nrx also seems to be part of transcriptional regulation, because it enhances the induction of the three transcription factors CREB (cAMP response element-binding protein), NF*κ*B (nuclear factor kappa B), and AP-1 (activator protein-1) [9]. In addition, Nrx was shown to regulate both the Wnt/PCP (planar cell polarity) and the Wnt/*β*-catenin pathway [10, 11]. The latter is inhibited through Nrx by binding to the basic PDZ domain of dishevelled (Dvl), thereby suppressing the redirection of the Wnt-induced signaling. This process is redox-dependent, because reducing conditions strengthen and oxidizing conditions weaken this protein-protein interaction [10]. Nrx might not only suppress this pathway but also retain a pool of inactive Dvl by preventing its proteasomal degradation. Binding of Nrx to the PDZ domain of Dvl prevents the possible interaction of Dvl and kelch-like protein 12 (KLHL12), which is part of an E3 ubiquitin ligase complex and leads to the ubiquitination and thus degradation of Dvl [12]. This mechanism may ensure that the pathway can be rapidly activated upon Wnt stimulation.

Here, we present the first comprehensive analysis of potential Nrx interaction partners and regulated pathways using a combination of two different strategies. First, we compared the redox state of the proteome from the human neuroblastoma cell line SH-SY5Y with cells in which the expression of Nrx was silenced. The redox state of the thiol proteome was identified and quantified using a differential thiol-labeling approach followed by quantitative mass spectrometry. Second, we utilized the unique reaction mechanism of Trx proteins to perform an intermediate trapping experiment using a Cys-Pro-Pro-Ser active site mutant of Nrx. With this approach, we trapped potential substrates from the SH-SY5Y cell line, as well as from murine brain tissue. We identified more than 50 proteins by all three approaches. Our findings imply potential functions of Nrx as an oxidase, rather than a reductase, in redox regulation of, e.g., metabolic pathways, cellular morphology, and signal transduction.

## 2. Material and Methods

### 2.1. Chemicals and Reagents

Chemicals and enzymes were purchased from Sigma-Aldrich (St. Louis, USA), unless otherwise stated, and were of analytical grade or better. Cell culture media and supplements were purchased from PAN-Biotech (Aidenbach, Germany) unless otherwise stated. Antibodies detecting Nrx (16128-1-AP, Proteintech, Manchester UK), actin (sc-47778, Santa Cruz Biotechnology, Dallas, USA), Prx1 (LF-MA0214, AbFrontier, Seoul, South Korea), Prx2 (serum, produced and validated by AG Lillig [5]), and tyrosine hydroxylase (Millipore MAP318) were used, as well as horseradish peroxidase-conjugated anti-rabbit and anti-mouse IgGs (Bio-Rad, Hercules, USA). SDS PAGE and Western blotting kits and equipment were purchased from Bio-Rad (Hercules, USA).

### 2.2. Cloning, Mutagenesis, Protein Expression, and Purification

The open reading frame of Nrx was amplified by PCR from mouse cDNA using the oligonucleotides 3′catatgtcgggcttcctggag5′ and 5′ggatccactagatgggctcaggc3′. Following A-tailing, the PCR product was ligated into the pGEM-T vector (Promega, Madison, USA) and was further subcloned by restriction ligation into the expression plasmid pET15b (Novagen, Darmstadt, Germany). For the intermediate trapping experiments, the more C-terminal active site cysteinyl residue of mouse Nrx was exchanged for a seryl residue (changing the Cys-Pro-Pro-Cys active site to Cys-Pro-Pro-Ser) by site-directed mutagenesis as described before in [13] using specific oligonucleotides (3′gtgtccacccagccgaagcc5′ and 5′taaggcttcggctgggtggac3′). Following the sequence analysis, the plasmid was transformed into the *E. coli* strain BL21(DE3)pRIL. Mouse Nrx Cys208Ser was expressed as a polyhistidine-tagged fusion protein in *E. coli* and was purified using the immobilized metal affinity chromatography technique and FPLC (ÄKTAprime, GE Healthcare, Uppsala, Sweden) as described before in [5]. The expression and purification efficiency was analyzed by SDS PAGE.

### 2.3. Cell Culture and Cell Transfection

SH-SY5Y cells were cultured in MEM medium without L-glutamine (PAA/GE Healthcare) supplemented with 2 mM L-glutamine and HeLa cells in DMEM at 37°C and 5% CO_2_ in a humidified atmosphere. Both media were supplemented with 10% FCS and 0.1 mg/ml streptomycin/100 U/ml penicillin.

Cells were transiently transfected with specific siRNA against Nrx (Eurogentec, Liège, Belgium; test siRNAs: Ambion, Carlsbad, California) (siNrx A sense: GGAUGACAUGACUGACUCCtt, antisense: GGAGUCAGUCAUGUCAUCCtc; siNrx B sense: GGCCUUUGUGAAUGACUUCtt, antisense: GAAGUCAUUCACAAAGGCCtc; siNrx C sense: GCCGAUAGCUGAGAAAAUCtt, antisense: GAUUUUCUCAGCUAUCGGCtg), as well as unspecific, scrambled control siRNA (sense: CAUUCACUCAGGUCAUCAGtt, antisense: CUGAUGACCUGAGUGAAUGtt) using electroporation as described before [14]. In brief, 5 · 10^6^ Mio SH-SY5Y cells or 3.5 · 10^6^ HeLa cells were resuspended in 550 *μ*l electroporation buffer (21 mM HEPES, 137 mM NaCl, 5 mM KCl, 0.7 mM Na2HPO4, 6 mM D-Glucose, pH 7.15), mixed with 15 *μ*g siRNA and transfected using 230 V, 500 Ω, and 1050 *μ*F for SH-SY5Y cells or 250 V, 500 Ω, and 1500 *μ*F for HeLa cells. The cells were mixed with 550 *μ*l prewarmed FCS and seeded in conditioned medium. After 72 h, cells were transfected a second time with the corresponding siRNA. After another 72 h, the cells were harvested using trypsin and were lysed as follows. For Western blot analysis and the 2-Cys Prx redox blot, the proteins were alkylated for 30 min at 37°C with 100 mM N-ethyl maleimide (NEM) (Pierce, St. Leon-Rot, Germany) in PBS prior to lysis and were then lysed for 30 min at room temperature in 2% CHAPS lysis buffer containing 5 mM NEM (40 mM HEPES, 50 mM NaCl, 1 mM EDTA, 1 mM EGTA, 1-fold protease inhibitor). For the intermediate trapping, cells and also the brain tissue were lysed in NP40 lysis buffer (10 mM Tris, 10 mM NaCl, 3 mM MgCl_2_, 0.1% NP40, pH 7.4).

### 2.4. SDS PAGE and Western Blot

The protein concentration of the clarified lysates was determined using the Bradford reagent (Bio-Rad, Hercules, USA). The proteins were diluted in TE buffer (10 mM Tris, 1 mM EDTA, pH 8) and mixed with sample buffer. 20-40 *μ*g proteins were separated by reducing (100 mM DTT) or nonreducing SDS PAGE for 30 min at 200 V using a 4-20% Mini PROTEAN TGX stain-free gel, which was subsequently activated according to the manufacturer's protocol, using the ChemiDoc XRS+ System. The proteins were transferred to a PVDF membrane using the Trans-Blot Turbo RTA Transfer Kit, according to the manufacturer's instructions. Using the ChemiDoc, the transferred proteins were imaged. This picture was used to normalize the Western blot signals to the total protein amount of the sample separated during the SDS PAGE. The membrane was blocked and incubated overnight with the specific primary antibody. The membrane was washed and incubated with HRP-coupled secondary antibody. The membrane was incubated with SuperSignal West Pico/Femto (Thermo Fisher Scientific, Waltham, USA) according to the manufacturer's instruction, to allow the detection of the resulting chemiluminescence with the ChemiDoc system. Western blots were densitometrically analyzed using ImageJ. In the case of the 2-Cys Prx redox blot, cells were treated with NEM prior and during cell lysis, and clarified lysates were subjected to nonreducing SDS PAGE and Western blot using specific antibodies against Prx1 and Prx2. The redox state of Prxs was quantified using ImageJ. The ratio of the reduced monomeric protein and the oxidized dimeric protein was analyzed. The 2-dimensional diagonal redox SDS PAGE was performed modifying the protocol described in [15]. In brief, 40 *μ*g cell lysate was mixed with sample buffer, denatured, and separated using a 4-20% PROTEAN TGX stain-free gel (Bio-Rad) at 200 V for 30 min. The protein lane was cut out and reduced in 250 mM DTT in sample buffer at 65°C. The proteins were alkylated for 20 min using 100 mM NEM in 1-fold sample buffer. Next, the gel was applied to a second 4-20% PROTEAN TGX IPG gel. A molecular weight marker was added, and the gel lane was overlayed with 1% agarose before the proteins were separated again. Following Western blotting, the total protein in the diagonal was visualized using the stain-free technology with the ChemiDoc XRS+ System. Pictures of the total protein in the diagonal (depicted in blue) and the specific protein of interest (in black) were overlayed using ImageLab 5.0 Software (Bio-Rad).

### 2.5. Immunocyto- and Histochemistry

Immunocyto- and histochemistry were performed as described in [5, 16]. The samples were analyzed with a Leica LCS SP2 confocal microscope (Leica, Wetzlar, Germany). Deconvolution and colocalization analysis was performed with the Huygens software package (Scientific Volume Imaging, Hilversum, Netherlands).

### 2.6. Intermediate Trapping and Mass Spectrometry

1.5 g CNBr-activated sepharose (GE Healthcare, Little Chalfont, UK) was prepared for coupling according to the manufacturer's protocol. 1.17 mg purified mNrx Cys208Ser was rebuffered with coupling buffer (0.1 M NaHCO_3_, 0.5 M NaCl, pH 8.3) using a PD-10 Sephadex G-25 column (GE Healthcare, Little Chalfont, UK) and added to the sepharose for 6 h at 4°C. After blocking the column overnight with 300 *μ*l blocking buffer (50 mM NaH_2_PO_4_, 1 mM ethanolamine, 1 mM HCl) and washing it with excess TE buffer, the protein was reduced adding 10 mM DTT in TE buffer (10 mM Tris, 1 mM EDTA, pH 8), followed by washing with TE buffer. After equilibrating the sepharose with 5 ml NP40 lysis buffer, 3 ml clarified SH-SY5Y lysates or mouse brain extracts were added to the column and incubated for 2 h at 4°C. Mouse brain extracts were isolated from Black 6 C57J mice, homogenized, and lysed in NP40 lysis buffer. The column was washed first with Tris buffer (100 mM Tris, 300 mM NaCl, pH 8) and then with TE buffer, before trapped proteins were eluted in 5 ml 10 mM DTT and 10 mM neutralized TCEP. A washing step with 3 ml TE buffer yielded a second eluate. The eluates were combined, and proteins were precipitated adding 10% TCA (final) (Roth, Karlsruhe, Germany). The proteins were pelletized by centrifugation (13300 rpm, 50 min, 4°C) and washed once with ice-cold 100% acetone (Roth, Karlsruhe, Germany) and twice with 80% (room temperature) acetone. Between the washing steps, the proteins were centrifuged for 45 min at 13300 rpm and 4°C. One part of the pellet was resuspended in 100 *μ*l urea buffer (8 M urea, 20 mM HEPES, 1 mM EDTA, pH 8) and separated using a Mini-PROTEAN TGX stain-free gel (Bio-Rad, Hercules, USA) at 100 V for one hour. The PageBlue (Fermentas, St. Leon-Rot, Germany) stained gel was analyzed via mass spectrometry, as well as a part of the pure, nonresuspended protein pellet. Supplementary Figure 2 shows a scheme depicting the intermediate trapping approach.

### 2.7. Differential Thiol-Redox Labeling with iodoTMT

Differential labeling was performed as described before in [17]. In brief, Nrx-depleted SH-SY5Y cells and scrambled siRNA control cells were harvested, washed with PBS, and lysed by sonication in UHE buffer (8 M urea, 20 mM HEPES, 1 mM EDTA, pH 8.0) containing one vial of iodoTMT™ labeling reagent, see Table 1 (Thermo Fisher Scientific, Waltham, USA), followed by incubation for 1 h at 30°C. The lysate was cleared, and proteins were acetone-precipitated. The precipitate was pelleted and washed with acetone, and the air dried pellet was dissolved, reduced, and alkylated in 100 *μ*l UHT buffer (8 M urea, 20 mM HEPES pH 8.0, 1 mM TCEP) containing a second vial of the iodoTMT™ labeling reagent, followed by incubation for 1 h at 30°C. The protein concentration was determined, equal protein amounts of three biological replicates were mixed, and proteins were acetone-precipitated. The pellet was washed with acetone, and the air-dried pellet was loosened in 25 mM ammonium bicarbonate buffer and digested for 2 h at 30°C adding trypsin at a protein to enzyme ratio of 100 : 1. The same amount of trypsin as before was added a second time, and the sample was incubated overnight at 30°C. The reaction was stopped by adding trifluoroacetic acid (TFA), and the proteins were freeze-dried. The pellets were dissolved in TBS, and the iodoTMT™ labeled peptides were purified using an anti-TMT™ antibody (Thermo Fisher Scientific, Waltham, USA). The peptides were eluted adding 400 *μ*l TMT (tandem mass tag) elution buffer (Thermo Fisher Scientific, Waltham, USA), followed by centrifugation. The supernatant was freeze-dried and dissolved in 5% acetonitrile/0.1% TFA. The samples were desalted via stage tipping. The peptides were separated with nano-HPLC and analyzed with an in-line coupled high-accuracy mass spectrometer. Spectra were analyzed with the computational proteomics platform MaxQuant with integrated quantification algorithms for chemical labels. Supplementary Figure 1 shows a scheme of the differential labeling approach.

The iodoTMT™ labels were also used to analyze changes in the abundance of proteins with cysteinyl residues. To do so, the samples were treated as described before except that the TCEP was already added to the lysis buffer with iodoTMT™ reagent to label all thiols in one step.

### 2.8. Functional Annotation Analyses

Gene ontology (GO) analysis and classification was done using the PANTHER data analysis and classification system. Proteins identified in the differential labeling approach with a *p* value < 0.05 and identified proteins from the intermediate trapping approaches with at least two determined iBAQs (intensity-based absolute quantification) were loaded separately into the classification tool. Proteins were analyzed for the ontologies' “biological process” and “molecular function” using a human background for the differential labeling and the intermediate trapping with SH-SY5Y extracts and a mouse background for the intermediate trapping data gained with mouse brain extracts.

## 3. Results and Discussion

Nrx is an active oxidoreductase that catalyzes thiol-disulfide exchange reactions *in vitro* [6]. It was shown to act as a signal transducer in some pathways, e.g., Wnt/Dvl [12]; however, no comprehensive analysis of its substrates and regulated pathways was available.

We applied two distinct strategies for the identification of Nrx targets. First, we performed a differential thiol-labeling approach that allows the specific analysis of the redox state of the whole thiol-redox proteome. We compared cells with siRNA-mediated silencing of Nrx expression to control cells treated with unspecific scrambled siRNA (see Supplementary Fig.  1). Second, we used an active site mutant of Nrx that lacks the C-terminal resolving cysteinyl residue within the dithiol active site motif. This protein was immobilized and allowed to react with potential target proteins. The thiol-disulfide exchange reaction mechanism of Trx family proteins requires the formation of an intermediate mixed disulfide of the N-terminal active site cysteinyl residue with the target protein. This intermediate is trapped in reactions with the mutant protein; hence, we named this approach intermediate trapping [18] (see Supplementary Fig.  2). We focused on redox-regulated targets in neuronal cells. Next to brain tissue from 6-week-old mice, we analyzed the cell line SH-SY5Y that was originally derived from a female patient with neuroblastoma. These cells display a high dopamine-*β*-hydroxylase activity and are able to form processes resembling dendrites and axons [19].

**Figure 1:**
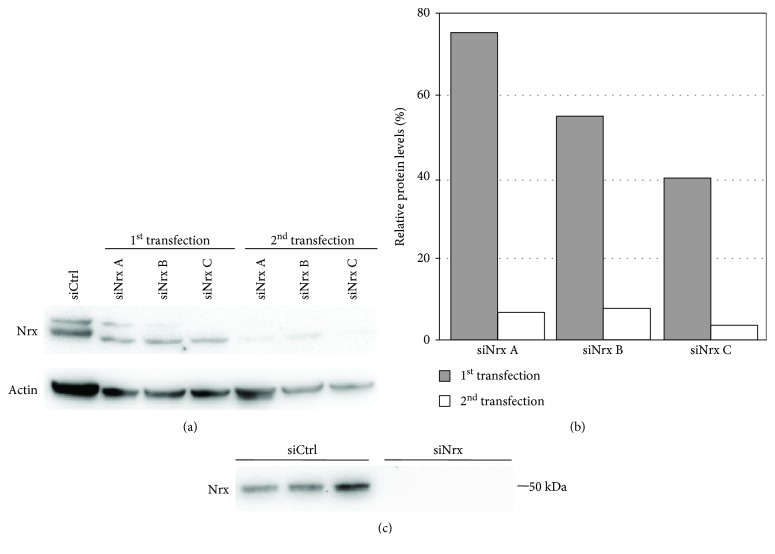
Establishment of siRNA-mediated gene-silencing in HeLa cells. Three different siRNAs against Nrx (siNrx A-C) as well as unspecific control siRNA (siCtrl) were used to establish a specific gene-silencing of Nrx. HeLa cells were transfected twice by electroporation, and 72 h after each transfection, cell extracts were prepared. The Nrx protein levels were analyzed by immunoblotting using specific antibodies against Nrx and actin (a). The quantification of the Western blot signals was performed using ImageJ and shows that siNrx C induced the most efficient knockdown of Nrx (b). Confirmation of the siRNA-mediated knockdown of Nrx in SH-SY5Y cells (*n* = 3) (c).

Nrx-specific RNA interference was established comparing three different siRNAs. All of them were effective in significantly reducing the levels of Nrx in HeLa cells (Figures 1(a) and 1(b)). The most effective one (siRNA C) reduced the levels of Nrx after consecutive rounds of transfections to below 3% compared to control-transfected cells and was used for further experiments. The efficiency of the knockdown was also confirmed in SH-SY5Y cells, where the protein was essentially undetectable following the siRNA treatment (Figure 1(c)).

**Figure 2:**
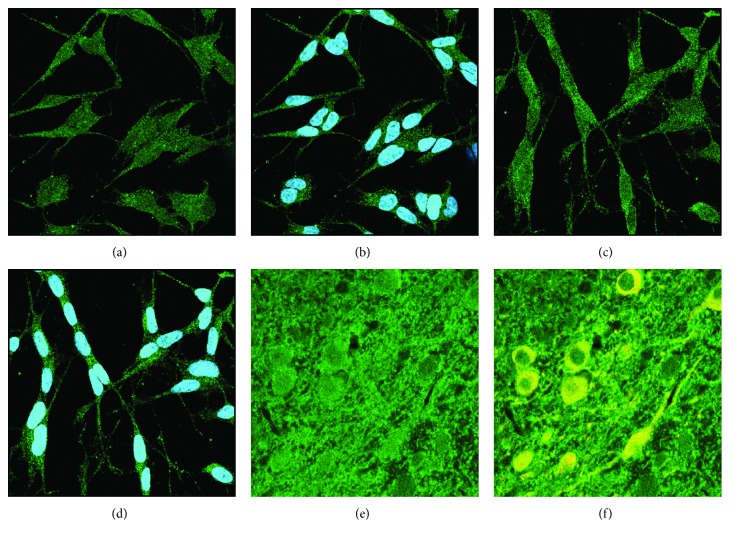
Subcellular localization of Nrx in SH-SY5Y cells and dopaminergic neurons of the mouse CNS. Immunocytochemistry stained for Nrx (green) and nuclei (blue in (b) and (d)) or tyrosine hydroxylase (yellow in (f)), analyzed by confocal microscopy. (a, b) SH-SY5Y control cells. (c, d) SH-SY5Y cells 72 hours after the beginning of neuron-like differentiation induced by retinoic acid. (e, f) Nrx staining in the substantia nigra of the mouse; dopaminergic neurons were labeled for tyrosine hydroxylase (yellow).

Nrx was described to act in both the nucleus [6] and cytosol, e.g., [12]. To clarify the localization of the protein in neuronal cells, we have analyzed the subcellular localization of the protein in both neuron-like SH-SY5Y cells and dopaminergic cells of the mouse substantia nigra by immunocyto- and histochemistry and confocal microscopy (Figure 2). In all analyzed cells, Nrx displayed a dual nuclear and cytosolic staining. This confirms previous results from our extensive analysis of redoxins in mouse tissues [5], as well as the data presented in the human protein atlas [20] (https://proteinatlas.org).

To analyze the thiol proteome in Nrx-depleted cells, SH-SY5Y cells were transfected and seeded in flasks for the labeling steps. For each condition, the siRNA-mediated gene-silencing of Nrx was confirmed by Western blot analysis (not shown). The samples were subjected to the differential labeling as described and analyzed by quantitative mass spectrometry; for details, see the experimental procedures, Table 1, and Supplementary Fig.  1. To our surprise, 161, i.e., 94.2%, of the 171 proteins identified with significant changes of the thiol-redox state were more reduced in the samples lacking Nrx expression (Figure 2(a)). These proteins displayed changes in at least one cysteinyl-containing peptide. The full list of these proteins was included in the supplementary material. These unexpected results suggest that Nrx may be involved in the oxidation of these proteins *in vivo*. The oxidation of protein thiols under physiological conditions is still a mystery. Although hydrogen peroxide can facilitate this, the low rate constants of most protein thiols with H_2_O_2_ preclude that this reaction occurs *in vivo* [2, 21, 22]. This led to the proposal that thiol- and selenol-peroxidases may act as sensors or transmitters by transferring the disulfides that form within their active site following the reduction of peroxides to specific target proteins [21]. Thiol-disulfide exchange reactions are fully reversible by nature. The direction of these reactions is determined by thermodynamic constrains such as the redox potential. The velocity of the reactions, however, is determined by the activity of enzymes that catalyze the reactions [22]. In fact, recent reports demonstrated that the oxidation of various protein thiols in cells depends on the presence of active peroxiredoxins [15]. It is tempting to speculate about a catalytic function of Nrx in disulfide relay pathways. In fact, we identified Prx1 as an interaction partner of Nrx in this study.

**Figure 3:**
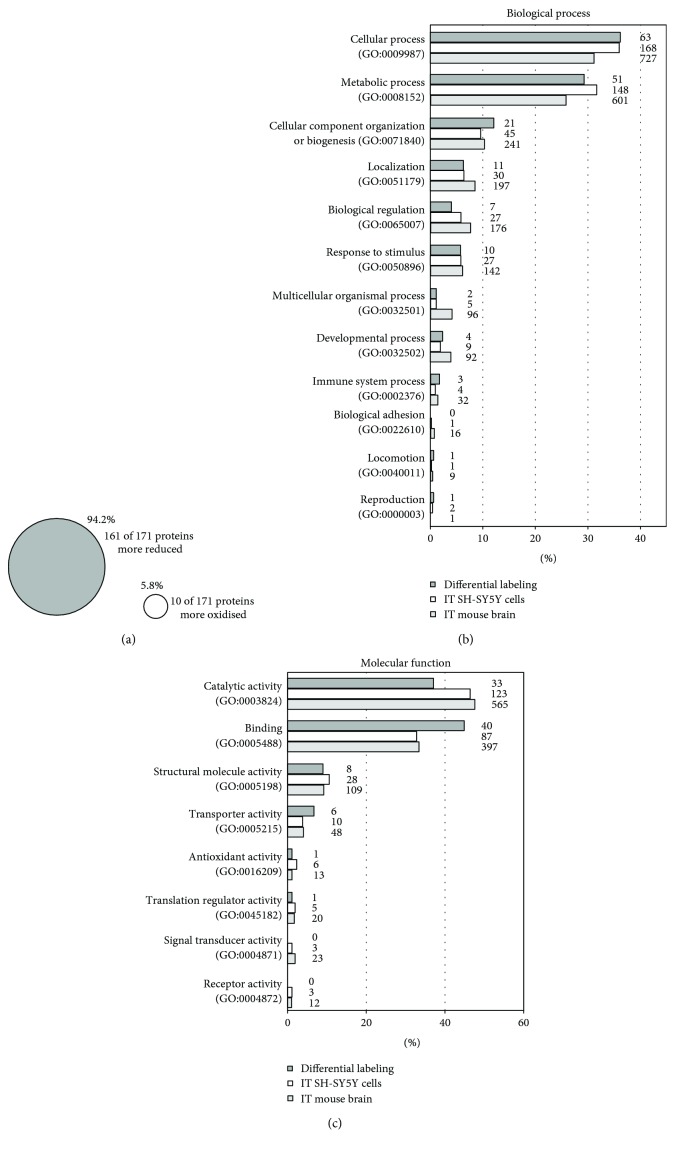
Classification of potential Nrx interaction partners identified by mass spectrometric analysis. To identify potential Nrx interaction partners and targets, three mass spectrometric-based approaches were performed, namely, a differential labeling with neuroblastoma SH-SY5Y cells lacking Nrx as well as two intermediate trappings (IT) with recombinant mNrx Cys208Ser using SH-SY5Y cell and mouse brain extracts. The results from the differential labeling were used to analyze the redox proteome of Nrx-depleted cells revealing that 94.2% of the identified cysteine-containing proteins were more reduced in the absence of Nrx (a). The potential interaction partners of Nrx, identified by all three approaches, were compared regarding their biological processes (b) and their molecular function (c) using the PANTHER classification system. There is a big resemblance between the results of the three approaches, with most potential interaction partners having catalytic activities and functions in binding, as well as metabolic and cellular processes.

To analyze the potential role of Nrx as oxidase of protein thiols also in cells of nonneuronal origin, we determined the redox state of both Prx1 and Prx2 in the HeLa cells lacking Nrx using Western blotting following nonreducing SDS PAGE (Figure 3). Prxs form an intermolecular disulfide in their reaction cycle that allows determining their redox state in samples treated with thiol alkylators during the harvesting and lysis of cells. Prx1, but not Prx2, was slightly more oxidized in the absence of Nrx, 11.1% compared to 7.2% in controls. Albeit statistical power was low (*p* value = 0.1, unpaired *t*-test analysis) (Figures 3(a) and 3(b)), the more oxidized Prx1 and the more reduced target proteins support the idea that Prx1 may oxidize Nrx in different cell types. Subsequently, Nrx transfers these oxidation equivalents to other proteins that cannot directly interact with the Prx.

Next to the differences in the redox state, the levels of 58 proteins were significantly altered, i.e., the *p* value of three independent samples was lower than 0.05. 30 of these proteins were decreased in the cells lacking Nrx compared to the control cells (see supplementary material).

For the second approach, we cloned mouse Nrx, produced the trapping mutant protein Cys208Ser by PCR, and purified the protein by metal affinity chromatography following recombinant expression in *E. coli* (see supplementary Figures 2 and 3). The proteins were immobilized and allowed to react with potential targets that were identified by mass spectrometry. The intermediate trapping of potential targets from mouse brain tissue as well as from the SH-SY5Y cell extracts yielded 1710 and 609 significant hits, respectively, in the databases. In contrast to the differential labeling approach, however, this approach does not allow concluding the direct or indirect reduction or oxidation of target proteins by Nrx due to the reversibility of the thiol-disulfide exchange reactions. The full lists of the identified proteins were also included in the supplementary material.

51 potential Nrx target proteins were identified by all three approaches and are listed in Table 2. Also, the analysis of the affected biological processes and molecular functions using the PANTHER data analysis and classification system [23, 24] yielded a high degree of overlap between the different experiments; see Figures 2(b) and 2(c). The lists are topped by cellular/metabolic processes and proteins with enzymatic activity, e.g., glyceraldehyde-3-phosphate dehydrogenase (GAPDH) or triose phosphate isomerase. These are followed by structural proteins, many of which function in a cellular component organization, e.g., cofilin 1, fascin 1, or kinesin light chain 1. Also, proteins with functions in the regulation of cellular processes and signal transduction were identified in significant numbers, for instance, guanine nucleotide-binding protein 1 or histone deacetylase 2.

**Figure 4:**
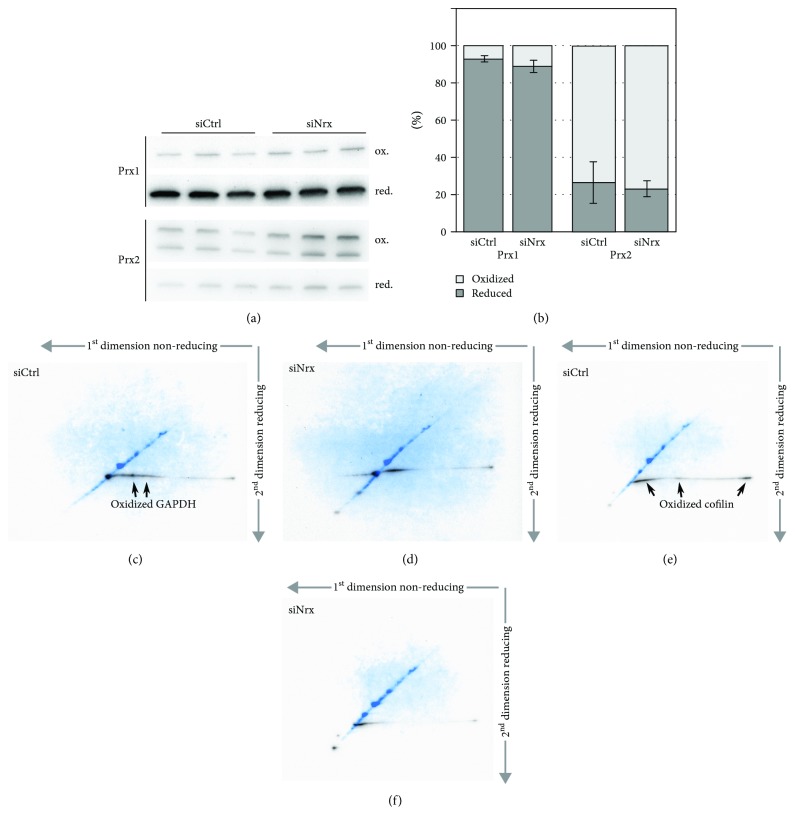
Redox state of cytosolic peroxiredoxins, cofilin, and GAPDH in Nrx-depleted HeLa cells. The redox state of Prx1 and Prx2 was analyzed in Nrx-depleted HeLa cells by 2-Cys Prx redox blot (a). Prior to lysis, free thiols were alkylated with NEM, and the monomer and dimer levels of the two proteins were analyzed via the 2-Cys Prx redox blot using specific antibodies against Prx1 and Prx2, respectively (a). The quantification of the Western blot signals using ImageJ (b) shows that more dimeric, i.e., oxidized Prx1, is present in the absence of Nrx (*p* value = 0.1). (c-f) Analysis of the redox state of potential Nrx targets by 2-dimensional diagonal gel electrophoresis and Western blotting. Both GAPDH and cofilin showed an increased staining of spots that fell below the diagonal in the second dimension in the presence of Nrx (c, e), compared to HeLa cells lacking Nrx (d, f). In the overlayed pictures, the total protein content in the diagonal is depicted in blue, the specific protein of interest in black.

Some of the proteins identified here as potential Nrx targets have been reported before to be regulated by the thiol-redox state. GAPDH, for instance, is inactivated by disulfide formation and other redox modifications of its active site Cys152 [25]. Here, we found this cysteinyl residue to be slightly more reduced (3.4%; *p* value 0.031) in the absence of Nrx in the cells of neuronal origin (see Supplementary Tables). We have also analyzed the redox state of this protein in the HeLa cell culture model using a 2-dimensional diagonal gel electrophoresis approach as described in [15] (Figures 4(c) and 4(d)). The protein was more oxidized in the presence of Nrx, as seen from the increased GAPDH staining below the diagonal (Figure 4(c)), compared to cells lacking Nrx (Figure 4(d)). Cofilin 1 was reported to be redox regulated by both glutathionylation [26] and intermolecular disulfide formation [27]. The protein seems to be inhibited by oxidation in its regulation of actin dynamics. Cell migration, for instance, seems to require the reduction of the protein [28]. We have confirmed the more reduced state of cofilin 1 also in the HeLa model using diagonal electrophoresis (Figures 4(e) and 4(f)). Nrx appears to promote the intermolecular disulfide-bonded form of cofilin 1 (Figure 4(e)). Hence, Nrx may, through the oxidation and inhibition of cofilin 1, negatively regulate cytoskelatal dynamics and cell motility.

One of the proteins with the most substantial changes in thiol-redox state was the triose phosphate isomerase. In the absence of Nrx, four cysteinyl residues were found to be more reduced: Cys67 (38.9%, *p* value 0.0385), Cys87 (29.4%, *p* value 0.0245), Cys127 (42.1%, *p* value 0.0125), and Cys218 (38.7%, *p* value 0.0479). The S-nitrosylation of the latter Cys218 residue has been reported to lead to a reduction in the activity of the human protein by 30% [29]. The glutathionylation of the human enzyme has been described in stressed T-lymphocytes [26]. In the close homologous enzymes from plants and algae, Cys127 and Cys218 can also be modified by glutathionylation [30] and other redox modifications (summarized, e.g., in [31]). The branched-chain amino acid aminotransferase was reported to be inactivated by S-nitrosylation and S-thiolation [32, 33]. In the absence of Nrx, we found the protein to be more reduced at Cys292 (20.1%, *p* value 0.044). Among the proteins identified here that were not yet reported to be regulated by redox modification of cysteinyl residues were the proteasomal subunit PSMA6 that was more reduced in the absence of Nrx at Cys47 (31.4%, *p* value 0.0238) and the dynein light chain 1 that was more reduced at Cys56 (23.8%, *p* value 0.0177). One of the few proteins that was significantly more oxidized in the absence of Nrx was the glutathione S-transferase omega 1 (13.4%, *p* value 0.0392) at the nonactive site residue Cys159.

We also identified proteins that were described as interaction partners of Nrx before. Nrxs suggested an electron donor in thiol-disulfide exchange reactions; the selenoprotein thioredoxin reductase 1 (TrxR1) [6] was identified in all three experiments. Protein phosphatase 2A was shown to form a stable complex with Nrx that may be important for the regulation of its activity [7]. In our differential labeling approach, protein phosphatase 2C A was one of the few proteins that was more oxidized in the presence of Nrx (Cys266, 6.6%, *p* value 0.0258). Interestingly, in plant cells, catalase is maintained in a reduced state by substrate-interaction with the Nrx homologue NRX1 [34].

Nrx has been implied in the redox regulation of cellular differentiation through the Wnt signaling pathway [10–12]. Our study suggests that it may also function in neuronal development. Reactive oxygen species and the redox state of various proteins contribute to neurogenesis, cell polarization, and maturation of neurons, providing a context for the spatiotemporal control of neural fate; see, for instance, [35]. The production of hydrogen peroxide is not sufficient to oxidize target proteins with significant rates [21, 22]. Nucleoredoxin may thus be a facilitator of hydrogen peroxide signaling by catalyzing the thiol-disulfide exchange reaction between peroxidases, such as Prxs, as sensors and the downstream target proteins that mediate the biological functions.

In summary, our study presented here suggests a number of specific functions for mammalian Nrx in the redox regulation of metabolic pathways, cellular morphology, and signal transduction pathways in neuronal cells. We identified numerous proteins with an altered thiol-redox state, dependent on the presence of Nrx. Astonishingly, most of these thiols were more reduced in the absence of this Trx family oxidoreductase. These results suggest a function of Nrx in the oxidation of these thiols, rather than their reduction. A possible way for the oxidation of Nrx itself may be the formation of disulfide relays with peroxiredoxins, supported by the direct interactions of the proteins demonstrated here.

## Figures and Tables

**Table 1:** 

Sample	1st label (reduced)	2nd label (oxidized)	Full label
siCtrl 1	126	129	126
siCtrl 2	127	130	127
siCtrl 3	128	131	128
siNrx 1	126	129	129
siNrx 2	127	130	130
siNrx 3	128	131	131

**Table 2:** Potential Nrx targets identified by all three approaches. The table contains the 51 proteins that were identified in all three experiments, i.e., the differential thiol labeling in Nrx-depleted SH-SY5Y cells, and the intermediate trapping using SH-SY5Y and mouse brain extracts. The proteins are sorted alphabetically; full lists can be found in the supplementary material.

Protein ID	Gene name	Protein name
P49588	AARS	Alanine-tRNA ligase, cytoplasmic
P23526	AHCY	Adenosylhomocysteinase
P49419	ALDH7A1	Alpha-aminoadipic semialdehyde dehydrogenase
P04075	ALDOA	Fructose-bisphosphate aldolase A
P48444	ARCN1	Coatomer subunit delta
P54687	BCAT1	Branched-chain amino acid aminotransferase, cytosolic
P78371	CCT2	T-Complex protein 1 subunit beta
P50990	CCT8	T-Complex protein 1 subunit theta
P23528	CFL1	Cofilin-1
Q00610	CLTC	Clathrin heavy chain 1
O94760	DDAH1	N(G),N(G)-Dimethylarginine dimethylaminohydrolase 1
P26641	EEF1G	Elongation factor 1-gamma
P13639	EEF2	Elongation factor 2
P09104	ENO2	Gamma-enolase
P21333	FLNA	Filamin-A
Q16658	FSCN1	Fascin
P04406	GAPDH	Glyceraldehyde-3-phosphate dehydrogenase
P62873	GNB1	Guanine nucleotide-binding protein
P17174	GOT1	Aspartate aminotransferase, cytoplasmic
P00505	GOT2	Aspartate aminotransferase, mitochondrial
Q92769	HDAC2	Histone deacetylase 2
Q99714	HSD17B10	3-Hydroxyacyl-CoA dehydrogenase type-2
P07900	HSP90AA1	Heat shock protein HSP 90-alpha
Q9NSE4	IARS2	Isoleucine-tRNA ligase, mitochondrial
P12268	IMPDH2	Inosine-5′-monophosphate dehydrogenase 2
Q07866	KLC1	Kinesin light chain 1
P28838	LAP3	Cytosol aminopeptidase
P55209	NAP1L1	Nucleosome assembly protein 1-like 1
P12955	PEPD	Xaa-Pro dipeptidase
P14618	PKM	Pyruvate kinase
P62937	PPIA	Peptidyl-prolyl cis-trans isomerase A
P53041	PPP5C	Serine/threonine-protein phosphatase 5
Q06830	PRDX1	Peroxiredoxin-1
Q9H6Z4	RANBP3	Ran-binding protein 3
P54136	RARS	Arginine-tRNA ligase, cytoplasmic
P39023	RPL3	60S ribosomal protein L3
P36578	RPL4	60S ribosomal protein L4
P61247	RPS3A	40S ribosomal protein S3a
Q16181	SEPT7	Septin-7
P37837	TALDO1	Transaldolase
P60174	TPI1	Triosephosphate isomerase
Q71U36	TUBA1A	Tubulin alpha-1A chain
Q9BVA1	TUBB2B	Tubulin beta-2B chain
Q16881	TXNRD1	Thioredoxin reductase 1, cytoplasmic
P62987	UBA52	Ubiquitin-60S ribosomal protein L40
P61088	UBE2N	Ubiquitin-conjugating enzyme E2 N
P45974	USP5	Ubiquitin carboxyl-terminal hydrolase 5
Q99536	VAT1	Synaptic vesicle membrane protein VAT-1 homolog
Q96QK1	VPS35	Vacuolar protein sorting-associated protein 35
P54577	YARS	Tyrosine-tRNA ligase, cytoplasmic
P63104	YWHAZ	14-3-3 protein zeta/delta (protein kinase C inhibitor protein 1)

## Data Availability

All data is available in the supplementary material.

## Abbreviations

Dvl: Dishevelled
GAPDH: Glyceraldehyde-3-phosphate dehydrogenase
H_2_O_2_: Hydrogen peroxide
NEM: N-Ethyl maleimide
Nrx: Nucleoredoxin
PDI: Protein disulfide isomerase
Prx: Peroxiredoxin
TMT: Tandem mass tag
Trx: Thioredoxin.

## Supplementary Materials

Supplementary 1 Supplementary Figure 1: scheme of the differential labeling approach. Supplementary Figure 2: scheme depicting the intermediate trapping approach used to identify potential Nrx interaction partners. Supplementary Figure 3: recombinant expression and purification of mouse Nrx WT and Cys208Ser in *E. coli*.

Supplementary 2 The list contains 171 potential Nrx interaction partners identified using the differential labeling approach in SH-SY5Y cells depleted of Nrx compared to control cells. Included are proteins with a significantly changed redox state, i.e., *p* values < 0.5. The proteins are in order of the differences in the thiol-redox state, beginning with the more reduced protein thiols.

Supplementary 3 This list shows 58 cysteine-containing proteins whose abundance is significantly changed (*p* value < 0.5) in SH-SY5Y cells lacking Nrx. The proteins were identified using the differential labeling approach with one iodoTMT™ label and a completely reduced proteome due to addition of the reductant TCEP.

Supplementary 4 Listed are 609 potential Nrx interaction partners that were identified with the intermediate trapping approach using mNrxC208S and SH-SY5Y extracts. Included are proteins, where at least two positive iBAQ (intensity-based absolute quantification) values were recorded. The iBAQ corresponds to the sum of all the peptide intensities divided by the number of observable peptides of a protein.

Supplementary 5 Using the intermediate trapping approach with mNrxC208S and mouse brain extracts, 1710 proteins with at least two positive iBAQ values were identified.
