# Supplementary material for: Nucleoredoxin-Dependent Targets and Processes in Neuronal Cells
Source: Oxid Med Cell Longev. 2018 Nov 21;2018:4829872. doi: 10.1155/2018/4829872 (PMC6280245; doi:10.1155/2018/4829872)

Supplementary figures to:

Nucleoredoxin-dependent targets and processes in neuronal cells

Urbainsky, Claudia[1], Noelker, Rolf[2], Imber, Marcel[3], Lübken, Adrian[1], Mostertz, Jörg[2], Hochgräfe, Falko[2], Hanschmann, Eva-Maria[1,4*], Lillig, Christopher Horst[1* #]

From the Institute for Medical Biochemistry and Molecular Biology, University Medicine, University of Greifswald, Germany (1); Competence Center Functional Genomics, Junior Research Group Pathoproteomics, University of Greifswald, Germany (2); Institute for Biology-Microbiology, Freie Universität Berlin, Germany (3), Department of Neurology, Medical Faculty, Heinrich-Heine University Düsseldorf, Germany (4)

* shared corresponding authors

Eva-Maria Hanschmann
Department of Neurology
Medical Faculty
Heinrich-Heine University Düsseldorf
Science Center
Merowinger Platz 1a
40225 Düsseldorf
Germany
Tel: +49 211 302039219
Eva-Maria.Hanschmann@med.uni-duesseldorf.de

Christopher Horst Lillig
Institute for Medical Biochemistry and Molecular Biology
University Medicine Greifswald
Ferdinand-Sauerbruch-Straße
DE-17475 Greifswald
Germany
Tel: +49 3834 865407
Fax: +49 3834 865402
horst@lillig.de

Supplementary Figure 1: Scheme of the differential labeling approach. Cells are harvested and lysed in the presence of an isobaric iodoTMT™ isomer (yellow circle) that binds to reduced thiol groups. IodoTMT™ label reagents are a set of isobaric isomers that are thiol-reactive and can be differentiated by mass spectrometry. Oxidized cysteinyl residues are reduced by TCEP. In a second labeling step the free thiols (formerly oxidized) are labeled with a different iodoTMT™ reagent (green circle). The samples can be analyzed by tandem mass spectrometry, to identify proteins with an altered redox state and to gain insights into the overall redox state of the proteome.

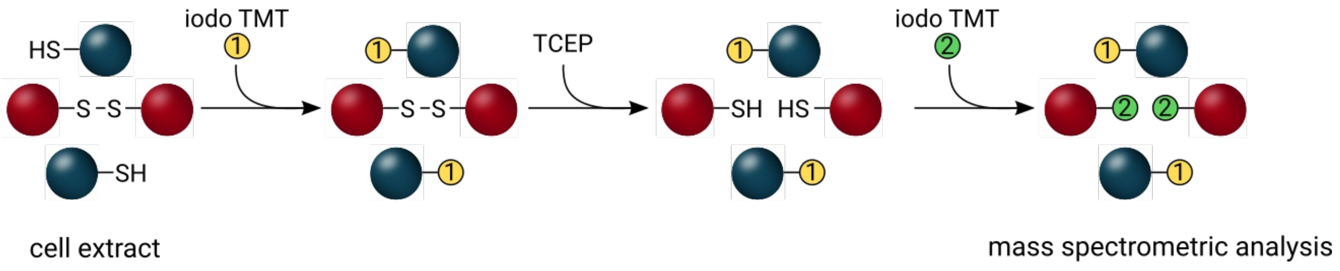

Supplementary Figure 2: Scheme depicting the intermediate trapping approach used to identify potential Nrx interaction partners. A: Recombinantly expressed and purified mNrx Cys208Ser protein lacking the resolving cysteine of the active site was linked to CnBr-Sepharose followed by reduction using DTT. Protein extracts from SH-SY5Y cells or mouse brain were loaded on the column. Potential interaction partners bound to the reduced Nrx, whereas unbound proteins were released in the flow-through. The trapped proteins were eluted by addition of the reductants DTT and TCEP. The eluat was analyzed by mass spectrometry, allowing the identification of potential Nrx interaction partners. B: Basis for this approach is the dithiol reaction mechanism of Trx family proteins (upper part), where the N-terminal active site cysteine attacks the disulfide bond of the oxidized target protein, leading to the formation of a mixed disulfide, which is reduced by the attack of the C-terminal active site cysteine, releasing the reduced target protein. The intramolecular disulfide within the active site of the Trx-family protein is reduced. Note, that the protein reducing Nrx is not known, yet. The exchange of the C-terminal active site cysteine for a serinyl residue (lower part) still allows the protein interaction with the target and the formation of the mixed disulfide, but prohibits the reduction and release of the target and therefore traps the intermediate.

A

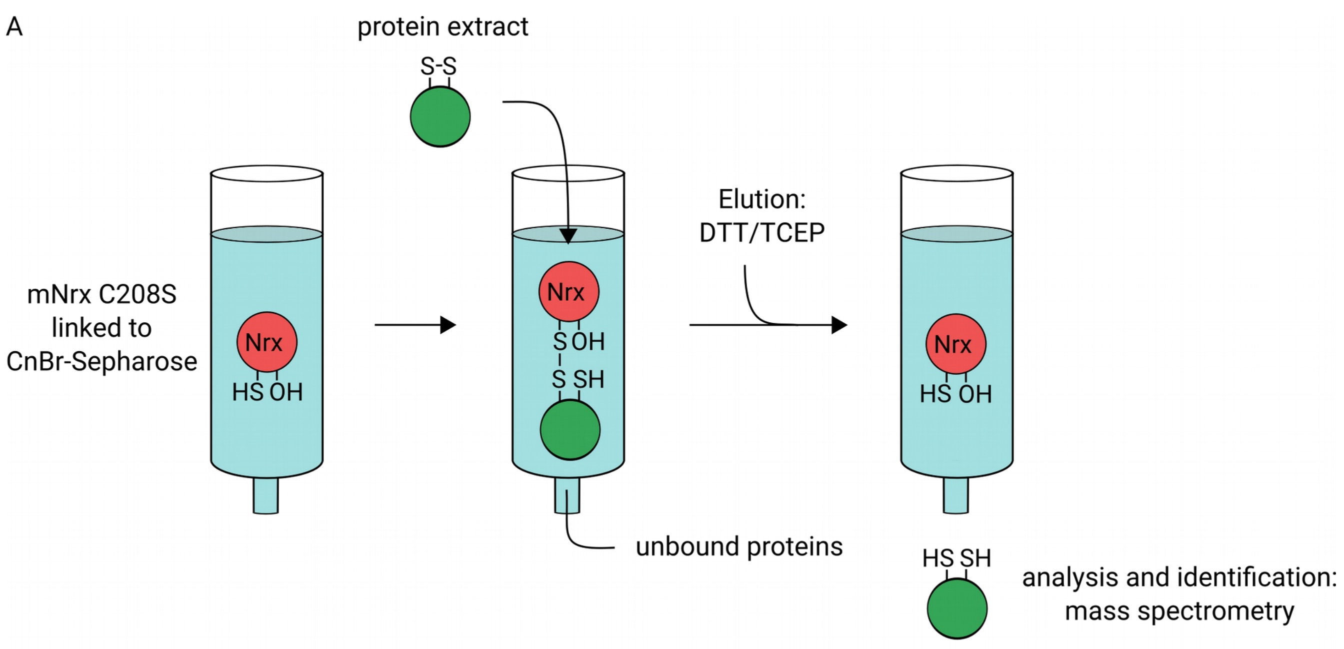
protein extract
S-S
mNrx C208S
linked to
CnBr-Sepharose
Nrx
HS OH
Nrx
S OH
S SH
Elution:
DTT/TCEP
Nrx
HS OH
unbound proteins
HS SH
analysis and identification:
mass spectrometry

B

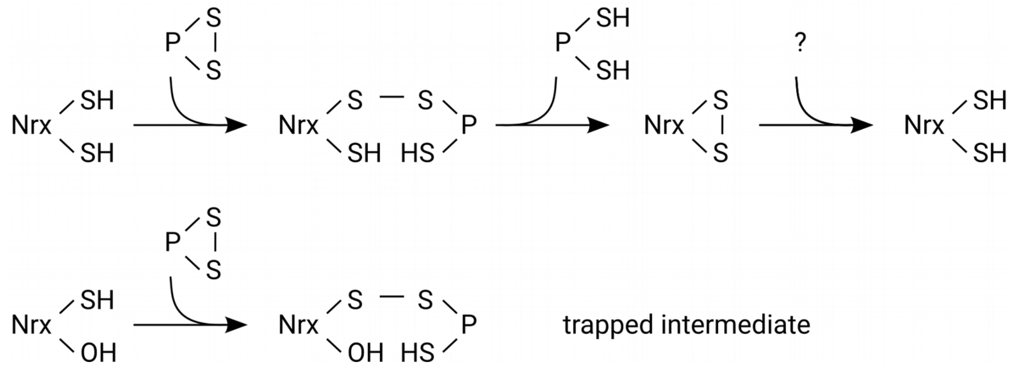
Nrx
SH
SH
P
S
S
Nrx
S
S
SH
HS
P
P
SH
SH
Nrx
S
S
?
Nrx
SH
SH
Nrx
SH
OH
P
S
S
Nrx
S
S
OH
HS
P
trapped intermediate

Supplementary Figure 3: Recombinant expression and purification of mNrx WT and Cys208Ser in *E. coli*. Proteins were expressed in *E. coli* BL21(DE3)pRIL and affinity-purified. The expression and purification was controlled by SDS PAGE. Samples before (- IPTG) and 5 h after induction (+ IPTG) of protein expression, as well as samples from the purification procedure, i.e. the extract of the lysed bacteria, the flow-through and the main fraction containing the purified His-tagged protein, confirmed a good yield and purity of recombinant mNrx protein.

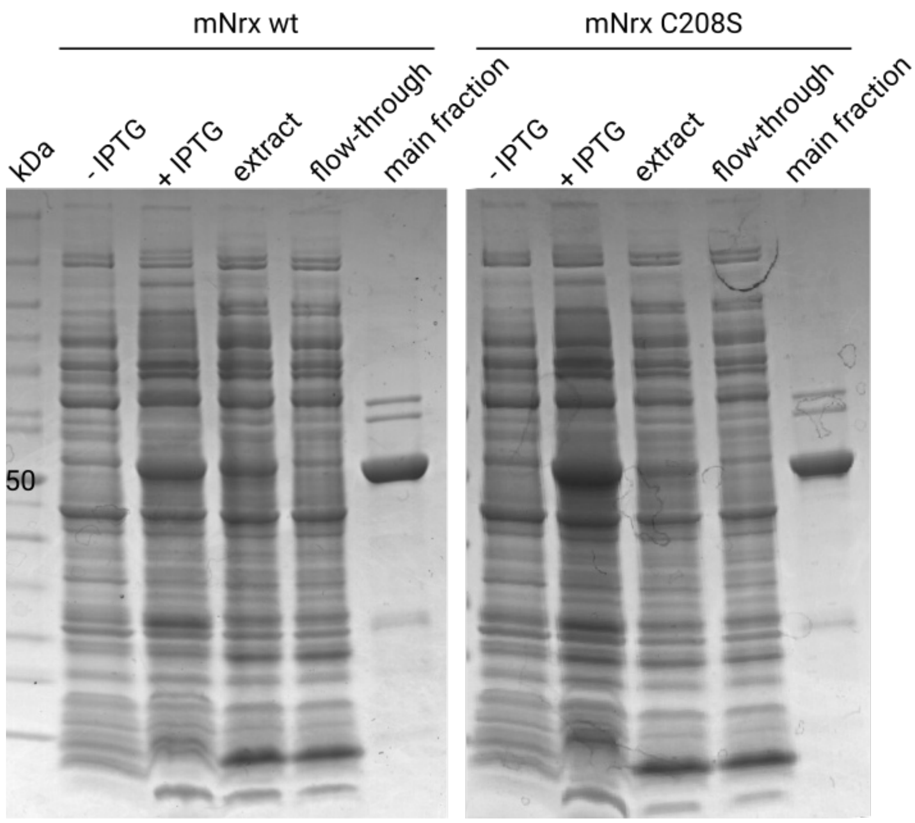

Supplement: Supplementary 1 — Supplementary Figure 1: scheme of the differential labeling approach. Supplementary Figure 2: scheme depicting the intermediate trapping approach used to identify potential Nrx interaction partners. Supplementary Figure 3: recombinant expression and purification of mouse Nrx WT and Cys208Ser in E. coli. [file 4829872.f1.pdf]
